# National Survey of Indigenous primary healthcare capacity and delivery models in Canada: the TransFORmation of IndiGEnous PrimAry HEAlthcare delivery (FORGE AHEAD) community profile survey

**DOI:** 10.1186/s12913-018-3578-8

**Published:** 2018-11-01

**Authors:** Jordan W Tompkins, Selam Mequanint, Douglas Edward Barre, Meghan Fournie, Michael E Green, Anthony J Hanley, Mariam Naqshbandi Hayward, Merrick Zwarenstein, Stewart B Harris, On behalf of the FORGE AHEAD Program Team, Stewart Harris, Ed Barre, Onil Bhattacharyya, David Dannenbaum, Keith Dawson, Roland Dyck, Jo-Ann Episkenew, Michael Green, Anthony Hanley, Barry Lavallee, Ann Macaulay, Alex McComber, Heather McDonald, Monica Parry, Sonja Reichert, Jon Salsberg, Braden TeHiwi, Amardeep Thind, Sheldon Tobe, Ellen Toth, Audrey Walsh, Jay Wortman, Lloy Wylie, Merrick Zwarenstein, Ross Bailie, Kayla Collins, Claire de Oliveira, Michael Hindmarsh, Valeria Rac, Joanne Lewis, Renee Bowers, Shubie Chetty, Brigitte Parent, Ratsamy Pathammavong, Lillian Houle, Amber Houle, Mary Jane Malcolm, Phyllis Racette, Sonya Houle, Dawn Montour-Lazare, Joelle Emond, Jessica Jacobs, Alexandra Audi, Randy Peterson, Randy Littlechild, Bonny Graham, Tina Littlechild, Ingrid Ekomiak, Devon Guy, Chalsea Onespot, Dawn Redmond, Kelsey Big Plume-Kahnapace, Ivan Kimble McComb, Emilie Dufour, Verna Jolly, Charlene Diamond, Mary Jacob, Stephanie Hester, Jennifer Jones, Danna Hadden, April DeYaeger, Theresa O’Keefe, Cynthia Benoit, Maggie Organ, Patricia Keesickquayash, Darlene Panacheese, Elaine Ishabid, Hazel Skunk, Edna Skunk, Marie Jebb, Carla Constant, Christie Wilson, Shelley Kirkness, Allen Deleary, Rennie Nawash, Lori Sinclair, Lisa Tabobondung, Melissa Gregory, Trudy Jacobs, Heather McDonald, Bonnie Nickel, Patricia Bobb, Kristina George, Jim Esler, Meghan Fournie, Jackie McLellan, Selam Mequanint, Kristina Miller, Mariam Naqshbandi Hayward, Jordan Tompkins, Marie Tyler, Susan Webster-Bogaert, Harsh Zaran

**Affiliations:** 1 Centre for Studies in Family Medicine, Western Centre for Public Health and Family Medicine, Department of Family Medicine, Schulich School of Medicine and Dentistry, Western University, 1511 Richmond Street, London, ON N6K 3K7 Canada; 2 Department of Health Sciences and Emergency Management, School of Professional Studies, Cape Breton University, Sydney, NS Canada; 3 Departments of Family Medicine and Public Health Sciences, Queen’s University, Kingston, ON Canada; 4 Departments of Nutritional Sciences and Medicine and the Dalla Lana School of Public Health, University of Toronto, Toronto, ON Canada

**Keywords:** National, Survey, Primary healthcare, Diabetes, Indigenous, Chronic disease, Quality improvement

## Abstract

**Background:**

There is a significant deficiency of national health information for Indigenous peoples in Canada. This manuscript describes the Community Profile Survey (CPS), a community-based, national-level survey designed to identify and describe existing healthcare delivery, funding models, and diabetes specific infrastructure and programs in Indigenous communities.

**Methods:**

The CPS was developed collaboratively through FORGE AHEAD and the First Nations and Inuit Health Branch of Health Canada. Regional and federal engagement and partnerships were built with Indigenous organizations to establish regionally-tailored distribution of the 8-page CPS to 440 First Nations communities. Results were collected (one survey per community) and reported in strata by region, with descriptive analyses performed on all variables. Results were shared with participating communities and regional/federal partners through tailored reports.

**Results:**

A total of 84 communities completed the survey (19% response rate). The majority of communities had a health centre/office to provide service to their patients with diabetes, with limited on-reserve hospitals for ambulatory or case-sensitive conditions. Few healthcare specialists were located on-site, with patients frequently travelling off-site (> 40 km) for diabetes-related complications. The majority of healthcare professionals on-site were Health Directors, Community Health Nurses, and Home Care Nurses. Many communities had a diabetes registry but few reported a diabetes surveillance system. Regional variation in healthcare services, diabetes programs, and funding models were noted, with most communities engaging in some type of innovative strategy to improve care for patients with diabetes.

**Conclusions:**

The CPS is the first community-based, national-level survey of its kind in Canada. Although the response rate was low, the CPS was distributed and successfully administered across a broad range of First Nations communities, and future considerations would benefit from a governance structure and leadership that strengthens community engagement, and a longitudinal research approach to increase the representativeness of the data. This type of information is important for communities and regions to inform decision making (maintain successes, and identify areas for improvement), strengthen health service delivery and infrastructure, increase accessibility to healthcare personnel, and allocate funding and/or resources to build capacity and foster a proactive chronic disease prevention and management approach for Indigenous communities across Canada.

**Trial registration:**

Current ClinicalTrial.gov protocol ID NCT02234973. Registered: September 9, 2014.

**Electronic supplementary material:**

The online version of this article (10.1186/s12913-018-3578-8) contains supplementary material, which is available to authorized users.

## Background

The chronic and progressive nature of diabetes has significant health and cost implications [1–4], and poses a substantial burden on patients, their families and communities, and the healthcare system. This burden is particularly pronounced in Indigenous populations, with type 2 diabetes mellitus (T2DM) prevalence rates 3 to 5 times higher in First Nations communities in Canada than the general population [5–13]. Complication rates associated with T2DM are also higher in First Nations populations, with rates estimated at 2 to 5 times higher than the general population [5, 13, 14]. There is an urgent need to shift diabetes incidence rates and timing of disease onset through transformative primary healthcare system redesign that enables effective care delivery capable of reducing diabetes-related complications and mortality for Indigenous peoples with T2DM [15–18]. This would include primary healthcare redesign to address the root causes of health inequities for Indigenous peoples through strengthening social determinants of health and recognizing the underlying history of colonization that is unequivocally linked to the health of Indigenous peoples [19–21], supporting leadership and self-governance of Indigenous peoples over their health [20–22], improving the patient experience through culturally sensitive patient-centered care, improving access to Indigenous primary healthcare and inter-professional healthcare providers [21], improving transitions between primary care and specialist care, and improving team based care and care coordination [23].

Strategies must also be designed to address the data challenge noted in the Truth and Reconciliations Calls to Action [21, 24], and reiterated by Smylie [25], highlighting the lack or inconsistency of Indigenous health information (datasets, surveillance systems or health surveys). This type of information is crucial to understanding the priorities and needs of a population, and thus informing health services, programs and best practices to direct health policy and funding [17, 26]. This data challenge has led to Indigenous peoples in Canada being “largely invisible in the majority of provincial and territorial health datasets” [25], and an inability to address health inequities across population groups in Canada. The First Nations Regional Health Survey (RHS) [22] was initiated as a pilot in 1997 due to the lack of First Nations and Inuit data in major national health surveys, and to acknowledge the need for Canada’s Indigenous peoples to self-govern their own health information. The RHS was designed with a longitudinal vision, with supplementary iterations in 2002/03 (Phase 1) and 2008/10 (Phase 2). Phase 3 of the RHS began its official roll-out in 250 First Nations communities in April, 2015 [27]. Governed by the First Nations Information Governance Centre (FNIGC), the RHS collects individual level data covering demographics, community wellness, early childhood, education, employment and income, health and well-being, housing, language and culture, nutrition and food security, and substance misuse and addictions. At the individual level, diabetes data includes the prevalence of chronic health conditions, health utilities index by prevalence of type 2 diabetes, type of diabetes (type 1, type 2, gestational), medication/treatment (for example, diet, pills, insulin, traditional medicine), and related complications (for example, hypertension, heart disease, glaucoma). To date, the RHS does not include community-level data related to the organization and availability of primary and specialist health services or providers, healthcare infrastructure, or programs for Indigenous peoples with diabetes. Recognizing the lack of Indigenous health data for informing primary healthcare redesign to address the increasing burden of diabetes and chronic disease in Indigenous communities, the Indigenous Primary Healthcare Capacity and Delivery Model Community Profile Survey (CPS) was developed as part of the TransFORmation of IndiGEnous PrimAry HEAlthcare Delivery (FORGE AHEAD) Research Program [28].

### FORGE AHEAD research program

FORGE AHEAD is a national research program that partners with First Nations communities from across Canada to improve chronic disease care and access to available resources by developing and evaluating community-driven, culturally-relevant primary healthcare models using quality improvement theory, tools and processes [28]. Over the 5 year program, activities have included an assessment of the current healthcare delivery, funding models and best practices used in First Nations communities in Canada [20], community and clinical readiness consultations to address and adopt chronic disease care [29], developing a community diabetes registry and web-based surveillance system [30] and evaluation and cost analysis of community and clinical quality improvement initiatives to improve chronic disease management. FORGE AHEAD aims to develop sustainable diabetes healthcare strategies and a scale-up toolkit for improved chronic disease management in Indigenous communities.

The purpose of this manuscript is to describe the development, and share the results of the CPS. The CPS was a national-level preparatory activity of FORGE AHEAD developed and implemented at the community level to identify and describe existing healthcare delivery, funding models, available infrastructure (nursing stations, healthcare centres, healing centres, hospitals), T2DM programs (primary prevention, screening, dialysis, etc.) and access and availability to healthcare professionals (nurses, physicians, diabetes educators, dietitians, etc.) and specialists (endocrinologist, optometrist, nephrologist, etc.) available to Indigenous communities across Canada. The survey was aimed at developing what is, to our knowledge, the first repository of public information of its kind of Indigenous communities across Canada.

## Methods

### Survey development and pilot testing

The CPS was developed through a collaborative partnership between FORGE AHEAD Indigenous community representatives, Western University research team members, and the First Nations and Inuit Health Branch (FNIHB) of Health Canada. Six First Nations communities involved in development of FORGE AHEAD were asked to pilot the survey in August 2013 to ensure clarity, comprehensiveness, relevance of the questions, language and terminology, length of the survey, and ease of completing the survey. Two communities responded from Manitoba and Québec, and all feedback was incorporated prior to distribution of the CPS. The final 8-page survey was available in English and French (Additional file 1). On average, the survey took 15–20 min to complete. The survey asked communities to complete the official community name, community address and population sizes, with instructions for one survey per community to be completed by the person most familiar with how healthcare was organized and operates (for example, Health Director, Nurse-in-charge or Band Council leader).

### Survey implementation

Prior to survey distribution, a list of all communities across Canada was obtained from the Indigenous and Northern Affairs Canada community profile website [31]. Community names were sent to regional offices for verification and the addition of community contact information. From this list, distribution of the CPS was planned for all 617 First Nations communities across Canada. Distribution of the CPS required building extensive collaborations with regional and national Indigenous organizations, including: FNIHB Health Canada national office, Saskatchewan (FNIHB regional office), Atlantic (FNIHB regional office covering Newfoundland, New Brunswick, Nova Scotia and Prince Edward Island), Québec (Cree Board of Health and Social Services of James Bay; First Nations of Québec and Labrador Health and Social Services Commission; Commission de la Santé et des Services Sociaux des Premières Nations du Québec et du Labrador, Ontario (Chiefs of Ontario), Manitoba (FNIHB regional office; Assembly of Manitoba Chiefs), Alberta (FNIHB regional office; FNIGC regional office), and British Columbia (First Nations Health Authority). Distribution to select sub-regions of Ontario and Alberta was not granted. Furthermore, concerted efforts were made to include communities in the Northern Region (Nunavut, Yukon and Northwest Territories); however, after careful review and consultation with representatives from this region, the on-reserve nature of the questions of the CPS were deemed not appropriate for distribution based on the differing governance and primary healthcare structures of communities in the region. Regional organizations were consulted and engaged as partners to develop region specific implementation strategies and to establish processes that complied with the Ownership, Control, Access and Possession (OCAP®) principles developed by the FNIGC [32]. These strategies included identifying and contacting all relevant organizations in the region that should be involved or made aware of the survey, identifying local trusted parties who could provide a letter of support to accompany the survey package, the details of the survey distribution strategy (initial contact, reminder and follow-up processes) and materials (survey tool, cover letter and letter of information from the principal investigator of FORGE AHEAD, letters of support and reminder letters). The goal was to ensure culturally appropriate and feasible approaches that considered the characteristics and contexts of each region, and promoted participation through the support of local trusted signatories.

Invitations to complete the survey were distributed by email, fax or mail. The survey was available to download and complete electronically as a fillable PDF or hard copy, and communities were provided a toll-free number to contact a member of the FORGE AHEAD research team for support. Completed surveys were returned via fax, email or postage-paid return envelope for communities that received a survey by mail. Three reminders were sent from Western University to all non-responding communities for a period lasting approximately 8 weeks, with telephone follow-ups to increase response rates. Participation in the survey was voluntary and did not require communities to participate in any other FORGE AHEAD activities.

### Ethical approval

Ethics approval for the FORGE AHEAD Program was granted by Western University Health Sciences Research Ethics Board (#103895, approved June 17, 2013), the Health Research Ethics Board of Alberta (CHC-14-0054, approved December 1, 2014), the Cree Board of Health and Social Services of James Bay (#2014-DSP-03, approved October 2, 2014), Mi’kmaw Ethics Watch, Unama’ki College, Cape Breton University (approved January 29, 2014), and Mi’kmaq Confederacy Ethics Review Committee, Prince Edward Island (approved March 14, 2014).

Health Canada ethics approval was not required because the CPS did not involve patients. Participation in the CPS was voluntary and completion of the survey indicated consent.

FORGE AHEAD is grounded in participatory research principles and approaches [33], and throughout the CPS, various processes were followed to honour OCAP® [32]. These included: community input on the content of the survey, community representatives on the FORGE AHEAD research team throughout the project, community level data belonging to the respective community with full access to their data (individual community results were not shared with other communities, agencies, etc.), only authorized team members had access to the survey data for research purposes, community receipt of individual data and a regional/national summary, and possession of the data remaining with each community and the FORGE AHEAD research team (data stored in a password-protected database and locked filing cabinet).

#### Analysis

Results were reported in strata by region and community size (small communities ≤300, medium > 300 to < 1500 and large ≥1500). Descriptive analyses were performed on all variables. Means, standard deviations, minimum and maximums were reported for all continuous variables. Number and proportions were reported for all dichotomous or categorical variables. The unit of analysis was the community. Results were summarized by sub-region (where applicable), region, and nationally. Community-specific reports were disseminated to all communities who completed a CPS highlighting community, regional (or sub-regional) and national level data. Regional and federal partners were provided aggregate data at the sub-regional/regional level and national level.

## Results

Between February 2014 and October 2014, the CPS was distributed to 440 of the 617 First Nations communities across Canada identified by Indigenous and Northern Affairs Canada [31]. Two surveys were distributed in March 2016 to communities participating in FORGE AHEAD as part of their involvement in the quality improvement program. As noted above, communities in the Northern Region and select sub-regions of Ontario and Alberta were excluded. A total of 84 communities completed the survey (19% response rate). All data was summarized to provide local community-level reports, including aggregated regional and national level information. Following report dissemination at the community level in April 2016, aggregate-level reports were provided to regional and federal partners.

Overall, 13.1% of communities were small with a population less than 300, 50.0% of communities were of medium size ranging from 300 to 1500, and 36.9% of communities were large, with more than 1500 people. The majority of communities surveyed (80.3%) were non-isolated, characterized by road access less than 90 km from the nearest physician services. Semi-isolated communities represented 12.4% of the communities surveyed, with 7.4% isolated or remote [34]. The average number of adults with T2DM per community was 164 (ranging from a minimum of 3 to a maximum of 1000). Table 1 provides a general overview of characteristics of the Indigenous communities across Canada that participated in the CPS.

**Table 1:** Community Characteristics, Healthcare Infrastructure & Resources of Indigenous Communities, Regionally and Nationally (*N* = 84)

	British Columbia *N* = 10	Alberta *N* = 7	Saskatchewan *N* = 15	Manitoba *N* = 18	Ontario *N* = 7	Québec *N* = 15	Atlantic *N* = 12	National *N* = 84
Community Characteristics								
Average Community Size, *n (range)*	1185 (200–3878)	4354 (250–15,223)	1579 (185–3900)	2312 (125–7933)	2610 (220–9109)	2102 (270–10,514)	724 (77–3192)	1972 (77–15,223)
Non-or semi-isolated communities (community has road access)^*^, *n (%*)	9 (90)	7 (100)	14 (93.3)	14 (77.8)	6 (85.7)	13 (86.7)	11 (91.7)	76 (90.5)
Healthcare Infrastructure^b^								
Nursing Station, *n (%)*	1 (10.0)	1 (14.3)	1 (6.7)	4 (22.2)	1 (14.3)	3 (20.0)	0 (0.0)	11 (13.1)
Health Office, *n (%)*	3 (30.0)	1 (14.3)	3 (20.0)	6 (33.3)	2 (28.6)	1 (6.7)	0 (0.0)	16 (19.0)
Health Centre, *n (%)*	9 (90.0)	7 (100)	15 (100)	11 (61.1)	6 (85.7)	11 (73.3)	11 (91.7)	70 (83.3)
Community Hospital, *n (%)*	2 (20.0)	7 (100)	0 (0.0)	0 (0.0)	0 (0.0)	1 (6.7)	0 (0.0)	3 (3.6)
No healthcare facility, *n (%)*	0 (0.0)	0 (0.0)	0 (0.0)	1 (5.6)	1 (14.3)	2 (13.3)	0 (0.0)	4 (4.8)
Resources								
Diabetes Registry, *n (%)*	5 (50.0)	4 (57.1)	3 (20.0)	8 (44.4)	1 (14.3)	10 (66.7)	3 (25.0)	34 (40.5)
Diabetes Surveillance System *n (%)*	2 (20.0)	2 (28.6)	2 (13.3)	6 (33.3)	0 (0.0)	4 (26.7)	2 (16.7)	18 (21.4)
Access to computer, *n (%)*	9 (90.0)	7 (100)	15 (100)	16 (88.9)	6 (85.7)	13 (86.7)	12 (100)	78 (92.9)
Access to internet, *n (%)*	10 (100)	7 (100)	15 (100)	16 (88.9)	6 (85.7)	13 (86.7)	12 (100)	79 (94.0)
Electronic medical record [EMR] for charting, *n (%)*	4 (40.0)	3 (42.9)	4 (26.7)	5 (27.8)	1 (14.3)	1 (6.7)	1 (8.3)	19 (22.6)

^a^First Nations and Inuit Health Branch. Available online: http://www.hc-sc.gc.ca/fniah-spnia/index-eng.php

^b^Communities were asked to select all that apply

### Healthcare infrastructure

Healthcare infrastructure and resources by region is presented in Table 1.

Communities were asked about the availability of healthcare facilities and resources in the community. Communities were instructed to select all that apply to their community; therefore, some communities may have selected more than one option. At a national level, 13.1% (*n* = 11) of communities have a nursing station, 19.0% (*n* = 16) have a health office (established by Indigenous organizations, governments or health programs within a community, services may not necessarily include patient care), 83.3% (*n* = 70) of communities have a health centre (primary location for patient care), and 3.6% (*n* = 3) had a community hospital. Four communities (4.8%) reported no healthcare infrastructure. When examining healthcare infrastructure by community size, 5.3% and 7.0% of small and medium sized communities had no healthcare infrastructure, while all large communities reported some type of healthcare infrastructure (nursing station, health office or health centre, community hospital). On a national level, 40.5% of the communities surveyed had a diabetes registry, and 21.4% had a diabetes surveillance system. Furthermore, in terms of electronic resources for the health facility, 92.9% reported having a computer, 94.0% reported having internet capabilities at the health facility, and 22.6% an electronic medical record for medical charting.

### Availability and access to healthcare professionals and specialists

Table 2 provides a breakdown of the availability of healthcare professionals and specialists by occupation at a national level. The majority of healthcare professionals were available on-site (57.1%), with 6.8% available indirectly (phone/internet), 11.9% available during regularly scheduled visits to the community, 14.7% available off-site ≤40 km away, and 9.6% available off-site > 40 km. For the availability and access to healthcare specialists, 4.6% of specialists (endocrinologist, nephrologist, etc) were available on-site, 13.5% available indirectly (phone/internet), 3.7% visiting, 16.5% available ≤40 km away, and 61.7% of healthcare specialists located off-site > 40 km away. The average number of on-site healthcare professionals per community was 9, while the average number of on-site healthcare specialists per community was 1.

**Table 2:** Availability and Access to Healthcare Professionals and Specialists at a National Level (*N* = 84)*

	On-Site (i.e., live and work in the community)	Indirectly available (i.e., through phone/internet, Telehealth)	Only Visiting (e.g., fly-in, mobile truck)	Available off-site (i.e., community members required to travel)	
				Off-site ≤40 km	Off-site > 40 km
Healthcare Professionals, *n (%)*					
Health Director	75 (89.3)	1 (1.2)	4 (4.8)	1 (1.2)	0 (0.0)
Nurse-in-charge	49 (58.3)	4 (4.8)	3 (3.6)	4 (4.8)	4 (4.8)
Family physician	27 (32.1)	8 (9.5)	19 (22.6)	22 (26.2)	13 (15.5)
Nurse Practitioner	12 (14.2)	0 (0.0)	8 (9.5)	18 (21.4)	6 (7.1)
Public Health Nurse	33 (39.3)	7 (8.3)	3 (3.6)	13 (15.5)	4 (4.8)
Community Health Nurse	67 (79.8)	2 (2.4)	6 (7.1)	4 (4.8)	5 (5.9)
Home Care Nurse	70 (83.3)	3 (3.6)	8 (9.5)	1 (1.2)	3 (3.6)
Community Health Representative	66 (78.6)	1 (1.2)	3 (3.6)	1 (1.2)	0 (0.0)
Personal Care Workers	62 (73.8)	1 (1.2)	2 (2.4)	2 (2.4)	0 (0.0)
Diabetes Nurse Educator	24 (28.6)	10 (11.9)	16 (19.1)	15 (17.9)	10 (11.9)
Community Diabetes Educator	36 (42.9)	5 (5.9)	5 (5.9)	6 (7.1)	4 (4.8)
Dietitian	25 (29.8)	7 (8.3)	16 (19.1)	18 (21.4)	14 (16.7)
Social Worker	41 (48.8)	1 (1.2)	4 (4.8)	12 (14.3)	5 (5.9)
Mental Health Therapist	32 (38.1)	10 (11.9)	22 (26.2)	12 (14.3)	7 (8.3)
Pharmacist	10 (11.9)	10 (11.9)	4 (4.8)	27 (32.1)	10 (11.9)
Dentist	19 (22.6)	3 (3.6)	8 (9.5)	20 (23.8)	16 (19.1)
Dental Hygienist	26 (30.9)	2 (2.4)	18 (21.4)	16 (19.1)	8 (9.5)
Traditional Healers / Elders	44 (52.4)	4 (4.8)	8 (9.5)	1 (1.2)	15 (17.9)
Cultural Coordinators	42 (50.0)	3 (3.6)	2 (2.4)	3 (3.6)	4 (4.8)
Specialists, *n (%)*					
Endocrinologist	3 (3.6)	12 (14.3)	1 (1.2)	9 (10.7)	56 (66.7)
Wound Care Specialist	6 (7.1)	19 (22.6)	0 (0.0)	11 (13.1)	48 (57.1)
Podiatrist/Chiropodist	7 (8.3)	10 (11.9)	3 (3.6)	12 (14.3)	53 (63.1)
Physiotherapist	10 (11.9)	8 (9.5)	3 (3.6)	30 (35.7)	8 (9.5)
Optometrist	6 (7.1)	8 (9.5)	8 (9.5)	26 (30.9)	42 (50.0)
Opthalmologist	3 (3.6)	9 (10.7)	9 (10.7)	13 (15.5)	53 (63.1)
Cardiologist/Internist	2 (2.4)	10 (11.9)	0 (0.0)	11 (13.1)	59 (70.2)
Nephrologist	1 (1.2)	10 (11.9)	0 (0.0)	7 (8.3)	62 (73.8)
Neurologist	1 (1.2)	10 (11.9)	1 (1.2)	9 (10.7)	58 (69.1)
Vascular Surgeon	1 (1.2)	11 (13.1)	1 (1.2)	9 (10.7)	61 (72.6)
Orthopedic	1 (1.2)	9 (10.7)	0 (0.0)	11 (13.1)	59 (70.2)
Plastic Surgeon	1 (1.2)	9 (10.7)	0 (0.0)	0 (0.0)	0 (0.0)
Psychiatrist	4 (4.8)	10 (11.9)	6 (7.1)	16 (19.1)	53 (63.1)
Pediatrician	4 (4.8)	10 (11.9)	8 (9.5)	14 (16.7)	53 (63.1)

* Unreported and Not Applicable responses were omitted from Table 2

### Healthcare services and diabetes programs of indigenous communities regionally and nationally

As is evident in Table 3, the majority of diabetes services and programs were available on-site, with the exception of dialysis and vaccinations (i.e. immunization clinics), where community members must regularly travel off-site (> 40 km). Community members must also regularly travel off-site for diabetes medications and labs, with over 60% of communities reporting these services off-site.

**Table 3:** National Availability and Access to Healthcare Services, Diabetes Programs and Supports (*N* = 84)

	On-Site (i.e., live and work in the community)	Indirectly available (i.e., through phone/internet, Telehealth)	Only Visiting (e.g., fly-in, mobile truck)	Available off-site (i.e., community members required to travel)	
				Off-Site ≤40 km	Off-site > 40 km
Dialysis, n *(%)*	1 (1.2)	3 (3.6)	2 (2.4)	16 (19.1)	54 (64.3)
DM care and management, *n (%)*	42 (50.0)	5 (5.9)	14 (16.7)	14 (16.7)	14 (16.7)
Medication, *n (%)*	23 (27.4)	3 (3.6)	3 (3.6)	33 (39.3)	16 (19.1)
Lab, *n (%)*	25 (29.8)	2 (2.4)	4 (4.8)	36 (42.9)	19 (22.6)
Vaccinations, *n (%)*	3 (3.6)	5 (5.9)	10 (11.9)	2 (2.4)	64 (76.2)
DM prevention, *n (%)*	64 (76.2)	4 (4.8)	9 (10.7)	9 (10.7)	2 (2.4)
Education and counselling for nutrition, *n (%)*	61 (72.6)	7 (8.3)	9 (10.7)	11 (13.1)	2 (2.4)
Mental healthcare, *n (%)*	39 (46.4)	8 (9.5)	20 (23.8)	15 (17.9)	15 (17.9)
Substance abuse, *n (%)*	68 (80.9)	3 (3.6)	5 (5.9)	9 (10.7)	8 (9.5)

Regional variation was reported for on-site diabetes clinical services and programs (ranging from 57.1% of communities in Ontario to 100% of communities surveyed in British Columbia and the Atlantic Provinces). Similar variation was noted in diabetes care, management and prevention, with less regional variation noted for diabetes education and counselling services (Table 4). Further exploration into innovative strategies designed to improve care for Indigenous peoples with diabetes on-site highlighted that at a national level, 61.9% of communities have diabetes training for healthcare providers/professionals, 75.0% of communities have designated diabetes health programs and interventions, 19.0% of communities are involved in diabetes health research projects, and 14.3% of communities have other innovative strategies to approach diabetes care that do not fit into the aforementioned categories (Table 5 provides a breakdown by region).

**Table 4:** On-Site Healthcare Services, Diabetes Programs and Supports, Regionally and Nationally

Diabetes Programs and Support	British Columbia *N* = 10	Alberta *N* = 7	Saskatchewan *N* = 15	Manitoba *N* = 18	Ontario *N* = 7	Québec *N* = 15	Atlantic *N* = 12	National *N* = 84
Clinical Services & Programs^a^, *n (%)*	10 (100.0)	7 (100.0)	11 (73.3)	12 (66.7)	4 (57.1)	13 (86.7)	10 (83.3)	67 (79.8)
Care, Management & Prevention^b^, *n (%)*	8 (80.0)	7 (100.0)	8 (53.3)	15 (83.3)	5 (71.4)	13 (86.7)	11 (91.7)	67 (79.8)
Education & Counselling^c^, *n (%)*	9 (90.0)	6 (85.7)	14 (93.3)	16 (88.9)	6 (85.7)	13 (86.7)	12 (100.0)	76 (90.5)

^a^Clinical Services & Programs: Dialysis treatment, Medication prescription, Laboratory services e.g. blood work, point of care testing, cultures, and Vaccinations e.g. immunization clinics

^b^Care, Management & Prevention: Diabetes care and management (treatment and screening of complications, e.g., foot care), and Diabetes prevention program e.g. awareness and screening

^c^Education & Counselling: Education & counselling for nutrition, healthy weight, physical activity, behaviour modification (e.g. smoking cessation), Mental healthcare including psychosocial counselling, Substance abuse awareness activities; counseling for addictions, Other

**Table 5:** Regional and National Innovative Strategies to Improve Care for Indigenous peoples with Diabetes

	British Columbia *N* = 10	Alberta *N* = 7	Saskatchewan *N* = 15	Manitoba *N* = 18	Ontario *N* = 7	Québec *N* = 15	Atlantic *N* = 12	National *N* = 84
Training for community healthcare professionals, *n (%)*	7 (70.0)	4 (57.1)	8 (53.3)	11 (61.1)	2 (28.6)	10 (66.7)	10 (83.3)	52 (61.9)
Designated community health programs and interventions, *n (%)*	8 (80.0)	7 (100)	11 (73.3)	12 (66.7)	4 (57.1)	10 (66.7)	11 (91.7)	63 (75.0)
Health research projects, *n (%)*	1 (10.0)	2 (28.6)	1 (6.7)	4 (22.2)	1 (14.3)	6 (40.0)	1 (8.3)	16 (19.0)
Other, *n (%)*	1 (10.0)	0 (0.0)	3 (20.0)	4 (22.2)	0 (0.0)	3 (20.0)	1 (8.3)	12 (14.3)

### Healthcare funding models

For communities with a healthcare centre, the average number of filled full-time equivalent (FTE) positions per community at a national level was 11.8 (ranging from a minimum of 0.4 and a maximum of 53.5, *n* = 58). Funding for healthcare providers on-site or visiting the community was predominantly provided by the federal government (41.8%), with additional 15.9% of provincial funding, 23.2% community, 3.8% tribal council and 15.7% of funding from ‘other’ sources.

Examining national pay structure, the majority (80.4%) of healthcare positions were paid on salary, 7.5% contract/per diem, 5.6% fee-for-service, 2.0% honorarium and 4.6% ‘other’ types of pay structures. Additional details by region regarding funding sources, pay structure, and filled FTE positions per community is provided in Table 6.

**Table 6:** Regional and National Funding Source and Pay Structure

	British Columbia *N* = 10	Alberta *N* = 7	Saskatchewan *N* = 15	Manitoba *N* = 18	Ontario *N* = 7	Québec *N* = 15	Atlantic *N* = 12	National *N* = 84
Funding Source								
Provincial	27.3%	0.0%	6.4%	8.5%	51.0%	25.2%	11.7%	15.9%
Federal	0.0%	28.1%	43.5%	70.6%	43.8%	44.8%	57.6%	41.8%
Community	50.4%	29.7%	42.9%	13.7%	1.0%	2.3%	29.8%	23.2%
Tribal Council	13.7%	0.8%	4.4%	3.4%	3.1%	3.5%	0.9%	3.8%
Other	8.7%	41.4%	2.9%	3.8%	1.0%	24.3%	0.0%	15.7%
Pay Structure								
Salary	42.5%	73.6%	81.6%	83.6%	63.6%	91.3%	91.4%	80.4%
Contract/Per diem	24.2%	11.7%	12.8%	0.2%	0.0%	4.4%	2.1%	7.5%
Fee-for-service	13.8%	5.3%	5.7%	11.3%	22.7%	1.2%	1.1%	5.6%
Honorarium	0.0%	5.3%	0.0%	0.0%	0.0%	3.2%	0.1%	2.0%
Other	19.5%	4.0%	0.0%	4.9%	13.6%	0.0%	5.3%	4.6%
Filled Full-Time Equivalent Positions per Community								
Average (min-max)	10.2 (4.6–31.3) *n* = 9	25.6 (8–53.4) *n* = 5	7.9 (2.0–15.5) *n* = 11	7.4 (0.4–19.0) *n* = 9	15.8 (9–28) *n* = 3	16.3 (6–31.2) *n* = 9	9.0 (0.5–24) *n* = 12	11.8 (0.4–53.5) *n* = 58

## Discussion

The goal of the CPS was to establish a unique and central repository of information on healthcare delivery, funding models, available infrastructure (nursing stations, healthcare centers, and hospitals) and healthcare professionals/specialists (part-time and full-time), and diabetes programs currently available in First Nations communities across Canada. A strong and accessible primary healthcare system is crucial to improving the health and health equity of a population [35], and the CPS survey and associated community and regional reports were designed to provide a current picture of healthcare delivery in each community, as well as region and nation. The results were designed to inform successes and to identify areas for improvement, and to assist communities and regions if a re-allocation of funding is needed to address care gaps for Indigenous peoples with diabetes. Results were also planned to inform primary healthcare redesign through the FORGE AHEAD quality improvement research program; however the timeline of data collection was delayed and results were not available during the design and implementation phase of the program. That being said, the results of the CPS, based on guidance from Indigenous communities involved in the program, may be incorporated into future plans for adapting and scaling up quality improvement programs like FORGE AHEAD, for broader scale adaptability and implementation across Canada.

The CPS was regionally tailored and distributed to 440 First Nations communities, culminating in development and distribution of 84 participating community reports and 51 regional/national reports to key stakeholders, including regional and federal government and organizational partners. Co-creation of the reports was extended to all Indigenous communities involved in the FORGE AHEAD research program and regional/national Indigenous organizations involved in this project so that the results were meaningful to communities and appropriately reflected the data [36, 37]. According to the CPS results, the majority of First Nations communities across Canada who completed the survey had a health centre or office to service their patient population with diabetes, with limited on-reserve hospitals for ambulatory or case sensitive conditions. Few healthcare specialists were located on-site, thus Indigenous peoples with diabetes frequently travel off-site (typically over 40 km) to see a specialist for diabetes-related complications. This has a detrimental impact on Indigenous peoples receiving timely and accessible care to optimize diabetes management and reduce long-term complications [15, 17]. The majority of healthcare professionals on-site were Health Directors, Community Health Nurses and Home Care Nurses, reinforcing the importance of continuing to put resources and funding into recruitment, retention and continuing education for these professionals who provide the majority of primary healthcare services. Many communities who completed the survey have diabetes registries, or a list of patients with diabetes; however, few have a surveillance system and method for tracking their patient population with diabetes (an essential component of high functioning primary healthcare systems) [17, 38, 39]. Regional variance in healthcare services, diabetes programs, and funding models was noted, with most communities engaging in some type of innovative strategy to improve care for their Indigenous patient population with diabetes. Saskatchewan reported the lowest percentage (53.3%) of communities with on-site diabetes care, management and prevention programs, while all communities in Alberta reporting on-site services. The majority of all regions reported diabetes education and counselling programs on-site, and further publications delineating regional variances and the potential impact of these services would be beneficial in the future. It is important to note that although these trends were observed for communities participating in the CPS, the low response rate means that the results should be interpreted with care, and should not be considered representative of all First Nations communities regionally or nationally across Canada. It is difficult to make a comparison of communities who responded to the survey versus communities who did not respond, or response rates due to a lack of similar regional or national-level Indigenous surveys to compare and contrast the CPS data. Recommendations for future research will seek to increase community engagement and the representativeness of the data as highlighted below, and work towards increasing Indigenous health data and understanding the characteristics and barriers faced by Indigenous people with diabetes across Canada.

### Recommendations for future research

Distribution of the CPS required extensive regional and federal engagement and partnerships with First Nations and other Indigenous organizations across the country. These partnerships were instrumental in establishing regionally-tailored distribution, ensuring regional or local Indigenous ethics review were obtained, and engagement at the community level. Future research with the CPS could advance the lessons-learned in this project to inform a longitudinal study design that transitions into a governance model that strengthens community engagement. Engagement and partnerships with regional and national Indigenous organizations such as the FNIGC, FNIHB, or other relevant organizations, with the goal of distribution and cultural tailoring to all Indigenous communities across Canada (including Métis and Inuit peoples), is important to increase the number of communities that respond and complete the survey, and thus optimize the representativeness of the data. Varying methods of survey distribution and data collection could be employed in future iterations of the CPS, including an online interface, expediting data entry and reducing data input errors. Increased response rates could also be facilitated by ensuring the survey is easy to complete, with questions relevant to the communities [40, 41], telephone follow-ups [41], and resources and personnel for future survey adaptation and implementation to optimize community responses. Revisions to the CPS could include a review of the survey questions to ensure clarity and comprehensiveness and cultural tailoring, including translation of the survey into Indigenous languages and adaptation for Inuit and Métis communities. Furthermore, questions could be added to examine whether communities are accredited for quality improvement work, as these communities may differ from communities not accredited [42], and identifying the total number of adults in the community over the age of 18 to calculate T2DM prevalence rates (the current CPS only asks for the total number of individuals of all ages living in the community). Lastly, future iterations of the CPS could include an analysis by community size or isolation level, and examine other determinants of variability to help understand geographic confounders that impact Indigenous health services and diabetes programs regionally and nationally.

The Truth and Reconciliation Calls to Action [21, 24] to establish measurable goals to identify and close the gaps in health outcomes between Indigenous and non-Indigenous communities clearly aligns with a longitudinal approach to the CPS. The CPS could assist over time in mitigating the deficiency of national health information for Indigenous peoples in Canada, and guiding regional or provincial/territorial strategic plans based on the needs of the population. Longitudinal CPS results would facilitate the data necessary to publish progress reports and establish long-term trends to determine if successes are being made for improved healthcare delivery and funding models (including culturally appropriate primary healthcare and healing practices), access and availability of Indigenous healthcare professionals and specialists (including retention of Indigenous healthcare providers), innovative programming for Indigenous people with diabetes, and the infrastructure to support these essential reform initiatives. As such, future versions of the CPS should be updated to capture critical information about the type and number of Indigenous healthcare providers, healing centers, and cultural competency training regionally and nationally to continuously inform primary healthcare reform initiatives in Indigenous communities across Canada. Continuous review and revisions of the design, collection and analysis of the CPS data with communities and regions would be beneficial for future research to ensure data is relevant, and that the appropriate governance, accountability, and control over Indigenous health information is upheld.

## Conclusion

Limited data are available on the healthcare delivery, funding models and diabetes programs available in Indigenous communities across Canada, and the CPS is the first community-based, national-level survey of its kind in Canada. Although the response rate was low, it is difficult to make a comparison due to a lack of similar national-level Indigenous surveys, and the CPS is one step towards mitigating the deficiency of national health information for Indigenous peoples in Canada. The CPS was distributed and administered successfully across a broad range of First Nations communities, and future considerations would benefit from a governance structure and leadership that strengthens community engagement, and a longitudinal research design fostering regional and national level data over time, to improve the representativeness of the data. This type of information is important for communities and regions to inform decision making (maintain what is being done well, and identify areas for improvement), strengthen health service delivery and infrastructure, respond more effectively to health service needs, increase accessibility to necessary healthcare personnel, and allocate funding and/or resources to build capacity and foster a proactive chronic disease prevention and management approach for Indigenous communities across Canada.

## Additional file

Additional file 1: FORGE AHEAD Community Profile Survey. (PDF 257 kb)

## Abbreviations

CPS: Community Profile Survey
FNIGC: First Nations Information Governance Centre
FNIHB: First Nations and Inuit Health Branch
FORGE AHEAD: TransFORmation of IndiGEnous PrimAry HEAlthcare Delivery
FTE: Full-time equivalent
OCAP®: Ownership, control, access and possession
RHS: Regional Health Survey
T2DM: Type 2 diabetes mellitus
