# Supplementary material for: National Survey of Indigenous primary healthcare capacity and delivery models in Canada: the TransFORmation of IndiGEnous PrimAry HEAlthcare delivery (FORGE AHEAD) community profile survey
Source: BMC Health Serv Res. 2018 Nov 1;18:828. doi: 10.1186/s12913-018-3578-8 (PMC6211407; doi:10.1186/s12913-018-3578-8)
Supplement: Supplementary file 1 — FORGE AHEAD Community Profile Survey. (PDF 257 kb) [file 12913_2018_3578_MOESM1_ESM.pdf]

Centre for Studies in Family Medicine • Schulich School of Medicine & Dentistry • Western University • Western Centre for Public Health & Family Medicine • 1151 Richmond St., • London, Ontario, Canada • N6A 3K7• t. 1-855-858-6872 ext. 2 (toll-free) • f. 1-877-809-5108 (toll-free)

COMMUNITY PROFILE SURVEY

GOAL: The Transformation of Indigenous Primary Healthcare Delivery (FORGE AHEAD) Research Program team invites you to participate in this national Community Profile Survey to learn more about the healthcare delivery, funding models, and diabetes programs currently available in First Nations communities. All First Nations communities across Canada are invited to participate in this survey, which is the first of many projects that are part of the five year FORGE AHEAD Research Program. FORGE AHEAD is designed with and for First Nations communities to improve care for people with diabetes. For more information about FORGE AHEAD, please visit our website at http://www.tndms.ca/forgeahead.html.

WHO SHOULD COMPLETE THIS SURVEY? The person who is most familiar with how healthcare is organized and operates in your community should fill in the survey. Typically, this can include the Health Director or Nurse-in-charge or band council leader. **Only one survey per community should be completed.** There are no right or wrong answers.

CONFIDENTIALITY: Survey responses are **confidential**. Community level data belongs to the respective community and the community will be given full access to their data. Only authorized team members will have access to the survey data for research purposes. Communities will receive the results of their individual data and a regional and national summary. Individual community results will NOT be shared with other communities, agencies, etc. The regional and national summary reports will not include names or other personal identifiers. Possession of the data will remain with the community and the FORGE AHEAD research team. Data will be stored in a password-protected database or stored in locked filing cabinet.

WHAT ARE THE BENEFITS? Communities can use the results of the survey to learn more about the current picture of healthcare delivery in their community, as well as in the region and nation. The results may help to inform decisions about maintaining what is being done well, to identify areas for improvement, and to assist communities and regions to re-allocate funding to address care gaps. The result will inform the design and implementation process of the rest of the FORGE AHEAD projects.

RISKS: There are no known risks to participating in this survey.

CONSENT: Participation in this survey is voluntary. You may refuse to participate and you may refuse to answer any questions. Completing the survey indicates your consent.

WHERE TO SEND YOUR COMPLETED SURVEY? Please send your completed survey to Meghan Fournie by scanning and attaching it to an **EMAIL** to **Meghan.Fournie@schulich.uwo.ca** or **FAX** (toll free) to **1-877-809-5108**.

CONTACT US: For questions about this survey, please contact Meghan Fournie (toll-free: **1-855-858-6872, ext #2**, email: Meghan.Fournie@schulich.uwo.ca). For questions about your participation in the study, please contact Dr. Stewart Harris (Principal Investigator), 519-858-5028. For questions about your rights as a research participant, or the conduct of the study, you may contact the Office of Research Ethics, 519-661-3036, ethics@uwo.ca.

SECTION 1: YOUR COMMUNITY PROFILE

Please complete your community's name and other information below.

1. **Community Name:** ______________________________

2. Address:

____

3. **Total** estimated community population:
   a) Total ____________
   b) Male ____________
   c) Female ____________

4. Estimated **on-reserve** population:
   a) Total ____________
   b) Male ____________
   c) Female ____________

5. Estimated **off-reserve** population:
   a) Total ____________
   b) Male ____________
   c) Female ____________

6. What is your community's isolation level?

   ☐ Non-isolated *(road access less than 90 km from nearest physician services)*
   ☐ Semi-isolated *(road access greater than 90 km from nearest physician services)*
   ☐ Isolated *(flights, good telephone service, no road access)*
   ☐ Remote isolated *(no scheduled flights, no road access, and minimal telephone and radio)*
   ☐ Don't Know

7. What type of health service centre(s) is available in your community? PLEASE MARK ALL THAT APPLY.

   ☐ Nursing Station
   ☐ Health Office
   ☐ Health Centre
   ☐ Community Hospital
   ☐ None: Go to Question #10

8. Are there any other communities (e.g. satellite communities) that are served by your community's health service centre(s)?

   ☐ Yes
   ☐ No

   If 'YES', please provide the names of each community served.

   ______________________________ ______________________________

   ______________________________ ______________________________

9. Does the health service centre(s) available in your community have any of the following? PLEASE MARK ALL THAT APPLY.

☐ Computer ☐ Internet access ☐ an electronic medical record (EMR) used for charting

10. Does your community have a searchable list of individuals with Type 2 diabetes (diabetes registry)?

☐ Yes ☐ No: Go to Question #14

11. When was the last time your community's diabetes registry was updated?

Month ________________ Year ____________________

12. What is the format of the diabetes registry in your community?

☐ Electronic-based ☐ Paper-based

13. Where is the community's diabetes registry housed?

☐ Within the Community ☐ Neighbouring Aboriginal Community ☐ Non-Aboriginal Urban Centre

14. Does your community have an electronic/computer system for tracking and monitoring patient's diabetes clinical information (diabetes surveillance system)?

☐ Yes ☐ No

15. What is the total estimated number of adults with type 2 diabetes in your community? ______________________

16. What is your current job title? ____

SECTION 2: HEALTHCARE PROFESSIONALS

1. Please check the appropriate box to indicate the **availability** (on-site, indirect, visiting, off-site) of **healthcare professionals** in your community. Where applicable, please provide the estimated **frequency of visits** for visiting healthcare professionals and **distance** for off-site healthcare professionals.

				**ONLY VISITING** (e.g., fly-in, mobile truck)		**ONLY AVAILABLE OFF-SITE** (i.e., community members required to travel)					
				**Estimated # of visits per (month OR year):**				**Estimated Distance:**			
**HEALTHCARE PROFESSIONALS**	**NOT APPLICABLE** (i.e., not available on- or off-site)	**USUALLY AVAILABLE ON-SITE** (i.e., live and work in the community)	**INDIRECTLY AVAILABLE** (i.e., through phone/internet, Telehealth)	month	year	**Neighbouring Aboriginal Community**	**Non-Aboriginal Town or Urban Centre**	<20km	20-40km	40-80km	>80km
Health Director	☐	☐	☐			☐	☐	☐	☐	☐	☐
Nurse-in-charge	☐	☐	☐			☐	☐	☐	☐	☐	☐
Family physician	☐	☐	☐			☐	☐	☐	☐	☐	☐
Nurse Practitioner	☐	☐	☐			☐	☐	☐	☐	☐	☐
Public Health Nurse	☐	☐	☐			☐	☐	☐	☐	☐	☐
Community Health Nurse	☐	☐	☐			☐	☐	☐	☐	☐	☐
Home Care Nurse	☐	☐	☐			☐	☐	☐	☐	☐	☐
Community Health Representative	☐	☐	☐			☐	☐	☐	☐	☐	☐
Personal Care Workers	☐	☐	☐			☐	☐	☐	☐	☐	☐
Diabetes Nurse Educator	☐	☐	☐			☐	☐	☐	☐	☐	☐
Community Diabetes Educator	☐	☐	☐			☐	☐	☐	☐	☐	☐
Dietitian	☐	☐	☐			☐	☐	☐	☐	☐	☐
Social Worker	☐	☐	☐			☐	☐	☐	☐	☐	☐
Mental Health Therapist	☐	☐	☐			☐	☐	☐	☐	☐	☐
Pharmacist	☐	☐	☐			☐	☐	☐	☐	☐	☐
Dentist	☐	☐	☐			☐	☐	☐	☐	☐	☐
Dental Hygienist	☐	☐	☐			☐	☐	☐	☐	☐	☐
Traditional Healers / Elders	☐	☐	☐			☐	☐	☐	☐	☐	☐
Cultural Coordinators	☐	☐	☐			☐	☐	☐	☐	☐	☐

2. Please check the appropriate box to indicate the **availability** (on-site, indirect, visiting, off-site) of **medical specialists** in your community. Where applicable, please provide the estimated **frequency of visits** for visiting specialists and **distance** for off-site specialists.

**MEDICAL SPECIALISTS**	**USUALLY AVAILABLE ON-SITE** (i.e., live and work in the community)	**INDIRECTLY AVAILABLE** (i.e., through phone/internet, Telehealth)	**ONLY VISITING** (e.g., fly-in, mobile truck)		**ONLY AVAILABLE OFF-SITE** (i.e., community members required to travel)					
			**Estimated # of visits per (month OR year):**		**Neighbouring Aboriginal Community**	**Non-Aboriginal Town or Urban Centre**	**Estimated Distance:**			
			month	year			<20km	20-40km	40-80km	>80km
Endocrinologist	☐	☐			☐	☐	☐	☐	☐	☐
Wound Care Specialist	☐	☐			☐	☐	☐	☐	☐	☐
Podiatrist /Chiropodist	☐	☐			☐	☐	☐	☐	☐	☐
Physiotherapist	☐	☐			☐	☐	☐	☐	☐	☐
Optometrist	☐	☐			☐	☐	☐	☐	☐	☐
Ophthalmologist	☐	☐			☐	☐	☐	☐	☐	☐
Cardiologist/Internist	☐	☐			☐	☐	☐	☐	☐	☐
Nephrologist	☐	☐			☐	☐	☐	☐	☐	☐
Neurologist	☐	☐			☐	☐	☐	☐	☐	☐
Vascular Surgeon	☐	☐			☐	☐	☐	☐	☐	☐
Orthopedic	☐	☐			☐	☐	☐	☐	☐	☐
Plastic Surgeon	☐	☐			☐	☐	☐	☐	☐	☐
Psychiatrist	☐	☐			☐	☐	☐	☐	☐	☐
Pediatrician	☐	☐			☐	☐	☐	☐	☐	☐
Other (specify): ______________	☐	☐			☐	☐	☐	☐	☐	☐
Other (specify): ______________	☐	☐			☐	☐	☐	☐	☐	☐
Other (specify): ______________	☐	☐			☐	☐	☐	☐	☐	☐

IF THERE IS NO HEALTH CARE CENTER AVAILABLE IN YOUR COMMUNITY, PLEASE GO TO SECTION 3 ON <u>PAGE 8</u>.

3. Please indicate the number of available and filled FTE positions, and employment/funding details of all healthcare providers that are **available <u>on-site or visiting</u>** for members of your community. Please DO NOT indicate peoples' names – only their positions. NOTE - *A FTE (Full-time equivalent) of 1.0 means that the person is a full-time worker; while a FTE of 0.5 means that the worker is half-time). Examples are provided below.*

Position	Number of available FTE positions	Number of filled FTE positions	Funding Source					Pay Structure				
			Provincial	Federal	Community	Tribal Council	Other (specify):	Salary	Contract/Per diem	Fee-for-Service	Honorarium	Other (specify):
Example: Nurse-In-Charge	*1.5*	*1*	☒	☐	☒	☐	☐	☒	☐	☐	☐	☐
Example: Dietitian	*1*	*0.2*	☐	☐	☒	☐	☐	☐	☒	☐	☐	☐
Family physician			☐	☐	☐	☐	☐	☐	☐	☐	☐	☐
Nurse-in-charge			☐	☐	☐	☐	☐	☐	☐	☐	☐	☐
Health Director			☐	☐	☐	☐	☐	☐	☐	☐	☐	☐
Nurse Practitioner			☐	☐	☐	☐	☐	☐	☐	☐	☐	☐
Public Health Nurse			☐	☐	☐	☐	☐	☐	☐	☐	☐	☐
Community Health Nurse			☐	☐	☐	☐	☐	☐	☐	☐	☐	☐
Home Care Nurse			☐	☐	☐	☐	☐	☐	☐	☐	☐	☐
Community Health Representative			☐	☐	☐	☐	☐	☐	☐	☐	☐	☐
Personal Care Workers			☐	☐	☐	☐	☐	☐	☐	☐	☐	☐
Community Diabetes Educator			☐	☐	☐	☐	☐	☐	☐	☐	☐	☐
Diabetes Nurse Educator			☐	☐	☐	☐	☐	☐	☐	☐	☐	☐
Dietitian			☐	☐	☐	☐	☐	☐	☐	☐	☐	☐
Social Worker			☐	☐	☐	☐	☐	☐	☐	☐	☐	☐

Position	Number of available FTE positions	Number of filled FTE positions	Funding Source					Pay Structure				
			Provincial	Federal	Community	Tribal Council	Other (specify):	Salary	Contract/Per diem	Fee-for-Service	Honorarium	Other (specify):
Mental Health Therapist			☐	☐	☐	☐	☐	☐	☐	☐	☐	☐
Pharmacist			☐	☐	☐	☐	☐	☐	☐	☐	☐	☐
Dentist			☐	☐	☐	☐	☐	☐	☐	☐	☐	☐
Dental Hygienist			☐	☐	☐	☐	☐	☐	☐	☐	☐	☐
Traditional Healers / Elders			☐	☐	☐	☐	☐	☐	☐	☐	☐	☐
Cultural Coordinators			☐	☐	☐	☐	☐	☐	☐	☐	☐	☐
Other, specify: ____________			☐	☐	☐	☐	☐	☐	☐	☐	☐	☐
Other, specify: ____________			☐	☐	☐	☐	☐	☐	☐	☐	☐	☐
Other, specify: ____________			☐	☐	☐	☐	☐	☐	☐	☐	☐	☐
Other, specify: ____________			☐	☐	☐	☐	☐	☐	☐	☐	☐	☐

SECTION 3: HEALTHCARE SERVICES AND DIABETES PROGRAMS

1. Please check the appropriate box to indicate the **availability** (on-site, indirect, visiting, off-site) of **healthcare services, diabetes programs and supports** in your community. Certain services may be available through multiple sources – PLEASE MARK ALL THAT APPLY. Where applicable, please provide the estimated **frequency of visits** for visiting services/program and **distance** for off-site services/programs.

**HEALTHCARE SERVICES AND DIABETES PROGRAMS**	**USUALLY AVAILABLE ON-SITE** (i.e., live and work in the community)	**INDIRECTLY AVAILABLE** (i.e., through phone/internet, Telehealth)	**ONLY VISITING** (e.g., fly-in, mobile truck)		**ONLY AVAILABLE OFF-SITE** (i.e., community members required to travel)					
			**Estimated # of visits per (month OR year):**		**Neighbouring Aboriginal Community**	**Non-Aboriginal Town or Urban Centre**	**Estimated Distance:**			
			month	year			<20km	20-40km	40-80km	>80km
**CLINICAL SERVICES & PROGRAMS**										
Dialysis treatment	☐	☐			☐	☐	☐	☐	☐	☐
Diabetes care and management (treatment and screening of complications, e.g., foot care)	☐	☐			☐	☐	☐	☐	☐	☐
Medication prescription	☐	☐			☐	☐	☐	☐	☐	☐
Laboratory services e.g. blood work, point of care testing, cultures	☐	☐			☐	☐	☐	☐	☐	☐
Vaccinations e.g. immunization clinics	☐	☐			☐	☐	☐	☐	☐	☐
Diabetes prevention program e.g. awareness and screening activities	☐	☐			☐	☐	☐	☐	☐	☐
**EDUCATION AND COUNSELLING**										
Education & counselling for nutrition, healthy weight, physical activity, behaviour modification (e.g. smoking cessation)	☐	☐			☐	☐	☐	☐	☐	☐

HEALTHCARE SERVICES AND DIABETES PROGRAMS	USUALLY AVAILABLE ON-SITE (i.e., live and work in the community)	INDIRECTLY AVAILABLE (i.e., through phone/internet, Telehealth)	ONLY VISITING (e.g., fly-in, mobile truck) Estimated # of visits per (month OR year):		ONLY AVAILABLE OFF-SITE (i.e., community members required to travel)					
					Neighbouring Aboriginal Community	Non-Aboriginal Town or Urban Centre	Estimated Distance:			
			month	year			<20km	20-40km	40-80km	>80km
Mental healthcare including psychosocial counselling	☐	☐			☐	☐	☐	☐	☐	☐
Substance abuse awareness activities; counseling for addictions	☐	☐			☐	☐	☐	☐	☐	☐
Other, specify: ____________	☐	☐			☐	☐	☐	☐	☐	☐
Other, specify: ____________	☐	☐			☐	☐	☐	☐	☐	☐

2. Has your community implemented **innovative strategies** related to the following activities that have changed **diabetes care in your community**? PLEASE MARK ALL THAT APPLY.

- ☐ Training for community healthcare providers/professionals
- ☐ Community health programs or interventions
- ☐ Health Research projects
- ☐ Other (specify): ____________________

Thank you very much for taking the time to complete this survey
